# Analysis of cognitive ability and adaptive behavior assessment tools used in an observational study of patients with mucopolysaccharidosis II

**DOI:** 10.1186/s13023-021-02118-3

**Published:** 2021-12-04

**Authors:** Karen S. Yee, Yanyu Wu, Magdalena Harrington, Susan E. Waisbren

**Affiliations:** 1 Takeda Development Center Americas, Inc., Cambridge, MA USA; 2 Shire (a member of the Takeda group of companies), Lexington, MA USA; 3 Present Address: Pfizer, 610 Main Street, Cambridge, MA 02139 USA; 4 Genetics and Genomics Division, Boston Children’s Hospital, Boston, MA USA

**Keywords:** Adaptive behavior, Cognitive ability, DAS-II, HS-FOCUS, Mucopolysaccharidosis II, VABS-II

## Abstract

**Background:**

Mucopolysaccharidosis II (MPS II) is a rare lysosomal storage disease characterized by cognitive impairment in most patients. This post hoc analysis evaluated changes in cognitive function, adaptive behavior and functional outcomes in patients with neuronopathic MPS II over time. Fifty-five children with MPS II were enrolled in a 24-month observational study (NCT01822184). The Differential Ability Scales, second edition (DAS-II; early years battery for ages 2 years 6 months to 6 years 11 months, school age battery for ages 7 years to 17 years 11 months), Vineland Adaptive Behavior Scales, second edition (VABS-II) and the Hunter Syndrome-Functional Outcomes for Clinical Understanding Scale (HS-FOCUS) were performed at baseline and 3-month intervals over 2 years. A subgroup of 38 children with a DAS-II General Conceptual Ability (GCA) score of 55–85 (below average–very low abilities) at any time during the study were included in this analysis.

**Results:**

Mean (standard deviation [SD]) early years DAS-II GCA score decreased from 73.4 (15.7, n = 22) at baseline to 62.7 (34.9, n = 6) at month 24. For the six patients with early years GCA assessments at baseline and month 24, mean (SD) GCA scores decreased from 72.3 (21.3) at baseline to 62.7 (34.9) at month 24. School age GCA scores were stable over 2 years: mean (SD) 72.4 (11.8, n = 10) at baseline; 74.3 (12.3, n = 8) at month 24. Mean (SD) VABS-II Adaptive Behavior Composite (ABC) scores were stable throughout the study (baseline, 81.8 [11.8, n = 36]; month 24, 81.0 [10.2, n = 13]). Some associations between items and domains of HS-FOCUS (*p* < 0.05) and DAS-II GCA and VABS-II ABC scores were shown, but there was no clear pattern of changes in HS-FOCUS over 2 years.

**Conclusions:**

The DAS-II measured changes in cognitive function over 2 years in younger patients with MPS II, whereas cognitive function in older patients remained stable. Further research is required to confirm the content validity of the DAS-II in different patient populations with MPS II. The VABS-II and HS-FOCUS were not sensitive tools for measuring behavioral and functional changes over 2 years. These findings may inform selection of appropriate cognitive and behavioral assessment tools for future studies.

**Supplementary Information:**

The online version contains supplementary material available at 10.1186/s13023-021-02118-3.

## Background

Mucopolysaccharidosis II (MPS II; Hunter syndrome; OMIM 309900) is a rare, X-linked, life-limiting lysosomal storage disease that typically affects males [1]. In affected patients, pathogenic variants in the *IDS* gene result in deficient activity of the lysosomal enzyme iduronate-2-sulfatase (I2S) [1]. This leads to accumulation of glycosaminoglycans in tissues and organs throughout the body, causing multiorgan dysfunction [1–3]. The presentation of MPS II and disease progression vary widely between patients [1–3]. Somatic clinical manifestations of MPS II include coarse facial features, hearing loss, severe airway obstruction, skeletal deformities, joint stiffness and cardiovascular disease [1–3]. The somatic burden of disease can be substantial and may severely impact patients’ physical functioning and their ability to perform day-to-day activities [4].

Approximately two-thirds of patients with MPS II have neuronopathic disease, in which they present with central nervous system involvement and cognitive impairment, in addition to somatic symptoms [1, 5, 6]. These patients typically experience a developmental plateau between the ages of 2 and 4 years, followed by deterioration of cognitive and adaptive behavior, decline in physical functioning and premature death, usually during the second decade of life [6, 7]. Patients with non-neuronopathic disease do not have central nervous system involvement, but may still have learning difficulties and behavioral problems as a result of somatic manifestations of the condition, such as hearing loss, impaired motor skills or brain abnormalities [8, 9].

The current standard of care for patients with MPS II is intravenous enzyme replacement therapy with recombinant I2S (idursulfase [Elaprase®]; Takeda Pharmaceuticals, Lexington, MA, USA) together with appropriate supportive symptomatic care [2, 10–13]. Enzyme replacement therapy with intravenous idursulfase has been shown to stabilize somatic symptoms of the disease [10–12]; however, it does not cross the blood–brain barrier in therapeutic quantities from intravenous delivery, and it is therefore not expected to affect neurological symptoms [14, 15].

Cognitive and adaptive behavioral testing can be used to evaluate disease progression, obtain a better understanding of the patient’s needs and assess the potential effects of interventional treatments [16–18]. Several tools have been used to assess cognitive and behavioral function in patients with MPS II. The Differential Ability Scales, second edition (DAS-II) and the Vineland Adaptive Behavior Scales, second edition (VABS-II) are comprehensive instruments that have been widely used to measure cognitive function and adaptive behavior, respectively [19, 20]. The Hunter Syndrome-Functional Outcomes for Clinical Understanding Scale (HS-FOCUS) is a parent/patient-reported instrument developed to measure limitations in physical functioning and daily activities specifically in patients with MPS II [4]. It has been validated using data from clinical trials of intravenous idursulfase and has good internal consistency, test–retest reliability and moderate to high correlation with other widely used health-related quality of life instruments [4, 21–23]. However, it is not known which components of these instruments best measure cognitive and adaptive behavioral decline in patients with MPS II. In addition, the association between HS-FOCUS scores and measurements obtained using the DAS-II and VABS-II has not been explored.

The neurodevelopmental and functional status of a pediatric population with MPS II was evaluated in a 2-year observational study using the DAS-II, VABS-II and HS-FOCUS. This post hoc analysis evaluated changes in all components of the DAS-II and VABS-II in patients with MPS II with evidence of early cognitive impairment over 2 years in the observational study, and assessed associations between measurements using these instruments and those obtained using HS-FOCUS. The aim was to better understand and interpret cognitive and adaptive function changes measured using these tools in patients with neuronopathic MPS II.

## Methods

### Study design

This post hoc analysis utilized data from a 24-month, multinational, multicenter, prospective, longitudinal, observational study (HGT-HIT-090; ClinicalTrials.gov identifier: NCT01822184). Patients were enrolled at sites in five different countries (Argentina, Mexico, Spain, the UK and the USA). The primary objective of the observational study was to evaluate the neurodevelopmental status of pediatric patients with MPS II over the course of 2 years.

The study was conducted in accordance with Good Clinical Practice, the International Council for Harmonisation of Good Clinical Practice Guidelines and the ethical principles of the Declaration of Helsinki. The protocol and informed consent documents were reviewed and approved by the institutional review board and/or independent ethics committee at each site participating in this study. Written informed consent was obtained for all patients from their parent(s) or legally authorized guardian(s) before any study-related procedures were conducted.

**Fig. 1:**
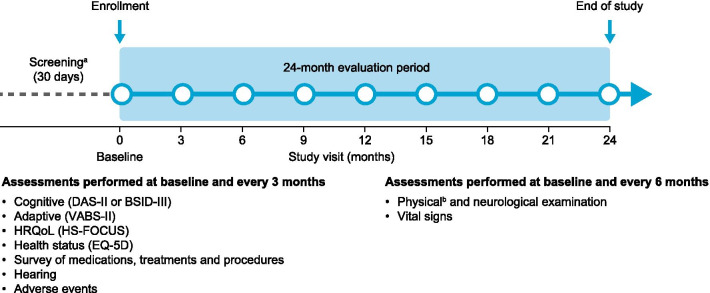
Study design. ^a^In addition to the assessments performed at baseline shown in the figure, the screening period includes: obtaining/evaluating informed consent/assent, inclusion/exclusion criteria, genotyping, demographic information and medical history. ^b^Height and weight were measured at baseline and at months 12 and 24 only. *BSID-III* Bayley Scales of Infant Development, third edition; *DAS-II* Differential Ability Scales, second edition; *EQ-5D* five-dimension EuroQol questionnaire; *HRQoL* health-related quality of life; *HS-FOCUS* Hunter Syndrome-Functional Outcomes for Clinical Understanding Scale; *VABS-II* Vineland Adaptive Behavior Scales, second edition

Enrolled patients attended the study site for assessments (including DAS-II, VABS-II and HS-FOCUS) at the following visits: screening (day − 30 to day − 1), baseline (day 0) and then at 3-month (± 14 days) intervals until the month 24 (± 14 days)/end of study visit (Fig. 1). If patients withdrew from the study or discontinued participation because of a parent or physician decision, assessments due to be conducted at month 24/end of study were carried out within 14 days of withdrawal/discontinuation. Primary assessments were cognitive function, using the DAS-II, and adaptive behavior, using the VABS-II. Secondary assessments included the HS-FOCUS, which measures functional outcomes. Neurodevelopmental testing was not repeated if it had been performed during the 8 weeks before the end of study visit.

### Patients

A total of 55 male patients aged 2– < 18 years with a confirmed diagnosis of MPS II and a DAS-II General Conceptual Ability (GCA) score of at least 55 (normative mean, 100; normative standard deviation [SD], 15) at screening were eligible for inclusion in the observational study which took place from January 2013 to October 2016. Patients enrolled in the natural history study therefore had a broad range of cognitive abilities at baseline. This post hoc analysis was performed using data from a subgroup of patients with early cognitive impairment, defined as a DAS-II GCA score of 55–85 (below average–very low abilities) at any time during the study. These patients were selected to evaluate the ability of the tools to measure changes in cognitive and adaptive function over time in patients with MPS II and cognitive impairment.

A diagnosis of MPS II was established by a deficiency in I2S enzyme activity of up to 10% of the lower limit of normal, as measured in plasma, fibroblasts or leukocytes, and either a documented pathogenic variant of the *IDS* gene that left the fragile X mental retardation 1 (*FMR1*) and 2 (*FMR2*) genes intact or a normal enzyme activity level of one other sulfatase, as measured in plasma, fibroblasts or leukocytes. All patients were required to have sufficient auditory capacity (with the use of hearing aids if required) to complete the study assessments. Patients receiving intravenous idursulfase as well as those not receiving idursulfase were eligible for enrollment. Key exclusion criteria were participation in an interventional trial at or during the 30 days before enrollment, or the presence of non-MPS II-related clinically significant central nervous system involvement, medical comorbidities or psychiatric comorbidities that could have affected administration, study data, integrity of the results or interpretation of protocol assessments.

Patients in this observational study could be enrolled in a phase 2/3 clinical trial evaluating intrathecal administration of idursulfase (HGT-HIT-094; ClinicalTrials.gov identifier: NCT02055118) if they met the eligibility criteria. The clinical decision to enroll eligible patients in the phase 2/3 trial was based on the judgment of the investigator. These patients then discontinued participation in the observational study.

### DAS-II, VABS-II and HS-FOCUS assessment tools

#### DAS-II

The DAS-II evaluates cognitive ability in children relative to same-age peers and consists of a series of tests administered by a qualified psychologist [19]. There are two test batteries; the early years test battery is designed for children aged 2 years 6 months to 6 years 11 months (with a lower level for ages 2 years 6 months to 3 years 5 months, and an upper level for ages 3 years 6 months to 6 years 11 months) and the school age test battery is designed for children aged 7 years to 17 years 11 months.

The DAS-II provides two composite scores (GCA and Special Nonverbal Composite), three cluster scores (verbal, non-verbal and spatial) and 10 core subtest scores, with higher values indicating better cognitive function. Composite and cluster scores are derived from different sets of subtests depending on whether patients are in the lower or upper level of the early years test battery or in the school age test battery (Table 1).

**Table 1:** Components of the DAS-II and VABS-II assessment tools

	DAS-II	VABS-II
Composite scores	GCA (overall score of cognitive performance) Derived from verbal and non-verbal cluster scores (all), and spatial cluster score (upper EY^a^ and SA only) SNC (similar to GCA but does not include verbal tests)	ABC (overall score of adaptive behavioral ability) Derived from all four domains for patients aged < 7 years and from three domains (motor skills excluded) for patients aged ≥ 7 years
Cluster/domain scores and component subtest/subdomain scores	Verbal Verbal comprehension (EY) Naming vocabulary (EY) Word definitions (SA) Verbal similarities (SA) Non-verbal Picture similarities (EY) Matrices (EY and SA) Sequential and quantitative reasoning (SA) Spatial Copying (EY) Pattern construction (EY and SA) Recall of designs (SA)	Communication Receptive Expressive Written Daily living skills Personal Domestic Community Socialization Interpersonal relationships Play and leisure time Coping skills Motor skills Gross Fine
Normative mean (SD)^b^	Composite and cluster scores: 100 (15)^c^ Subtest scores: 50 (10)^d^	ABC and domain scores: 100 (15) Subdomain scores: 15 (3)

*ABC* Adaptive Behavior Composite; *DAS-II* Differential Ability Scales, second edition; *EY* early years (2 years 6 months–6 years 11 months); *GCA* General Conceptual Ability; *SA* school age (7 years–17 years 11 months); *SD* standard deviation; *SNC* Special Nonverbal Composite; *VABS-II* Vineland Adaptive Behavior Scales, second edition

^a^The DAS-II early years test battery comprises a lower level for children aged 2 years 6 months to 3 years 5 months and an upper level for those aged 3 years 6 months to 6 years 11 months

^b^DAS-II composite/cluster scores and subtest scores are normed on different scales and ABC/domain scores and subdomain scores are also normed on different scales

^c^Composite scores were the sums of subtest *T* scores normed; cluster scores were the sum of two normalized subtest *T* scores

^d^Normalized *T* scores were used for all subtest scores

Subtest scores and cluster/composite scores are normed on different scales. This analysis used normalized *T* scores (mean 50, SD 10) for all subtest scores. Cluster scores (mean 100, SD 15) were derived from the sum of two normalized subtest *T* scores. The GCA composite score was derived from the sum of six subtest *T* scores and normed with a mean of 100 and an SD of 15.

#### VABS-II

The VABS-II is a standardized norm-referenced tool used to evaluate adaptive behavior in individuals from 0 to 90 years of age and is administered by a psychologist during a semi-structured interview with the patient’s parent or caregiver [20]. The VABS-II has one composite score (the Adaptive Behavior Composite [ABC]), which provides an overall measure of adaptive behavior ability. The ABC comprises four domain scores in patients younger than 7 years old (communication, daily living skills, socialization and motor skills) and three domain scores in patients 7 years of age or older (communication, daily living skills and socialization). The domain scores are derived from 11 subdomain scores (Table 1). The ABC and domain scores have a normative mean of 100, with an SD of 15. The subdomain scores (V scale) are normed with a mean of 15 and an SD of 3.

#### HS-FOCUS

The HS-FOCUS is an MPS II-specific tool used to evaluate functional status based on parent-/caregiver-completed and patient self-reported questionnaires [4, 21, 22]. A shortened version of the questionnaire developed to decrease respondent burden was used in this observational study [21]. The HS-FOCUS shortened parent-/caregiver-completed version assesses functional status in five domains (walking/standing, grip/reach, school/work, activities and breathing) comprised 21 items (reduced from 68 items in the original instrument) [21]. Each item is scored using the following response scale: 0 = with no difficulty, 1 = with some difficulty, 2 = with much difficulty, 3 = unable to do. Higher scores correspond to worse functional capacity. Self-report questionnaires were not used in this study because of the young age and low cognitive ability of many of the patients.

The shortened version of the HS-FOCUS has acceptable internal consistency reliability (Cronbach’s alpha > 0.70) and test–retest reliability (intraclass correlation coefficient for most domains > 0.70, the set criterion for good reliability), and moderate (> 0.3) to high (> 0.6) Spearman correlations with the Childhood Health Assessment Questionnaire, Childhood Health Questionnaire and Health Utilities Index [21, 23].

### Statistical methods

Graphs were plotted for mean DAS-II GCA and VABS-II ABC scores (± 95% confidence intervals [CIs]) over time. To ensure that measurement scales were comparable, the mean percentage change from baseline was also evaluated for all DAS-II and VABS-II subtest/subdomain, cluster/domain and composite scores (which are normed on different scales). Items in the HS-FOCUS were ordinal variables and stacked column plots were therefore produced to show changes in functional abilities over time.

A mixed-effects model for repeated measures (MMRM) was performed to identify associations between baseline variables (age, genotype, cognitive function and hearing impairment) and composite DAS-II GCA and VABS-II ABC scores measured over time for up to 2 years. The MMRM was also used to provide adjusted graphical plots for all components of the DAS-II and VABS-II (subtest/subdomain, cluster/domain and composite scores) and all items and domains of the HS-FOCUS to compare trends. There was no adjustment for alpha in these multiple group comparisons. In addition, the MMRM was used to evaluate associations between DAS-II GCA and VABS-II ABC scores and HS-FOCUS items, while controlling for within-patient correlations and any associated baseline variables. The purpose of these analyses was to establish any associated trends between measurements of cognitive ability, adaptive behavior and functional outcomes specific to MPS II. For the MMRM statement, compound symmetry was selected for the covariance structure because it provided a better model fit (based on the Akaike information criterion) [24] than an unstructured covariance structure.

## Results

### Study population

In total, 38 of the 55 patients in the observational study had evidence of early cognitive impairment as determined by a DAS-II GCA score of 55–85 at any time during the study and were included in this analysis. Most patients were younger than 7 years old (27/38, 71.1%) and their median (range) age at baseline was 4.90 years (2.11–16.74) (Table 2). *IDS* genotypes in order of frequency were missense/presumed missense (20/38, 52.6%) followed by presumed splice site/splice site (9/38, 23.7%), frameshift (3/38, 7.9%), nonsense (3/38, 7.9%), premature truncated (2/38, 5.3%) and large deletion (1/38, 2.6%). Of 32 patients with an available baseline DAS-II GCA score, 16/38 (42.1%) had a GCA score of 70 or lower, and the same proportion had a GCA score above 70. DAS-II GCA score at baseline was missing for 6/38 patients (15.8%). The mean (SD) baseline GCA score (including both early years and school age batteries) was 73.1 (14.4), indicating that cognitive function was low in this patient population (normative mean 100, SD 15). The mean (SD) baseline VABS-II ABC score was 81.8 (11.8), indicating moderately low adaptive function (normative mean 100, SD 15). Overall, 19 of the 38 patients (50%) included in this analysis discontinued participation in the observational study to enroll in the phase 2/3 intrathecal idursulfase trial. A further four patients discontinued participation from the observational study for other reasons. Patient flow through the study is shown in Additional file 1: Fig. S1.

**Table 2:** Patient baseline characteristics

Characteristic	Study population (N = 38)
Male, n (%)	38 (100)
Age, years	
Median (range)	4.90 (2.11–16.74)
Age subgroups, n (%)	
< 7 years	27 (71.1)
≥ 7 years	11 (28.9)
Race, n (%)	
Asian	2 (5.3)
African American	1 (2.6)
White	32 (84.2)
Other	3 (7.9)
Genotype category, n (%)	
Frameshift	3 (7.9)
Large deletion	1 (2.6)
Missense/presumed missense	20 (52.6)
Nonsense	3 (7.9)
Premature truncated	2 (5.3)
Presumed splice site/splice site	9 (23.7)
Baseline DAS-II GCA score^a^ subgroups, n (%)	
≤ 70	16 (42.1)
> 70	16 (42.1)
Missing	6 (15.8)
Baseline DAS-II GCA score^a^, n = 32	
Mean (SD)	73.1 (14.4)
Baseline VABS-II ABC score^b^, n = 36	
Mean (SD)	81.8 (11.8)

*ABC* Adaptive Behavior Composite; *DAS-II* Differential Ability Scales, second edition; *GCA* General Conceptual Ability; *SD* standard deviation; *VABS-II* Vineland Adaptive Behavior Scales, second edition

^a^Standard scores of ≤ 69, 70–79, 80–89 and 90–109 indicate very low, low, below average and average function, respectively

^b^Standard scores of ≤ 70, 71–85 and 86–114 indicate low, moderately low and adequate adaptive levels, respectively

### DAS-II

**Fig. 2:**
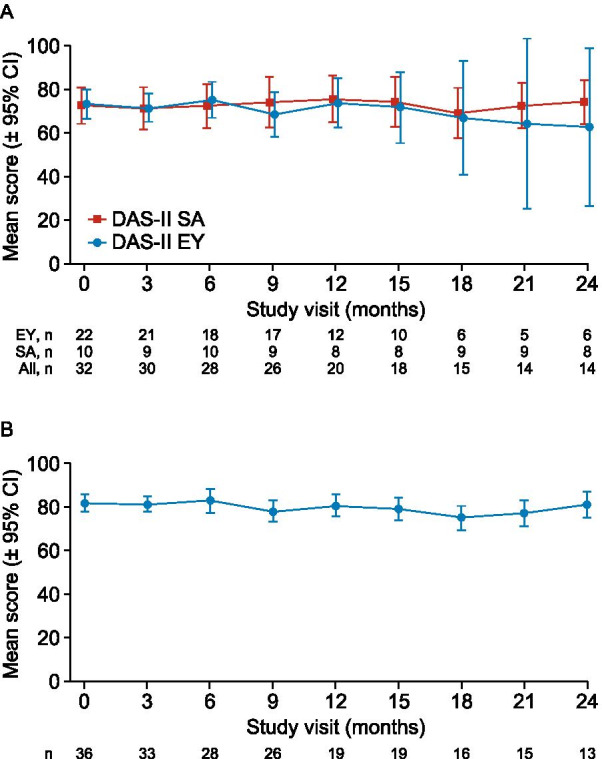
Mean DAS-II GCA and VABS-II ABC standard scores: **A** DAS-II early years and school age GCA scores; **B** VABS-II ABC scores. *ABC* Adaptive Behavior Composite; *CI* confidence interval; *DAS-II* Differential Ability Scales, second edition; *EY* early years (2 years 6 months–6 years 11 months); *GCA* General Conceptual Ability; *SA* school age (7 years–17 years 11 months); *VABS-II* Vineland Adaptive Behavior Scales, second edition

The mean (SD [95% CI]) DAS-II early years GCA score decreased throughout the study, from 73.4 (15.7 [66.4, 80.3], n = 22) at baseline to 62.7 (34.9 [26.1, 99.2], n = 6) at month 24 (Fig. 2). For the six patients with assessments available at both baseline and month 24, mean (SD [95% CI]) early years GCA score was 72.3 (21.3 [50.0, 94.7]) at baseline and 62.7 (34.9 [26.1, 99.2]) at month 24. Individual DAS-II GCA scores were also plotted for these patients (Additional file 1: Fig. S2), however no clear patterns were observed. The mean (SD [95% CI]) school age GCA score was 72.4 (11.8 [63.9, 80.9], n = 10) at baseline and 74.3 (12.3 [63.9, 84.6], n = 8) at month 24. Individual DAS-II GCA scores by visit are shown in Additional file 1: Table S1.

**Fig. 3:**
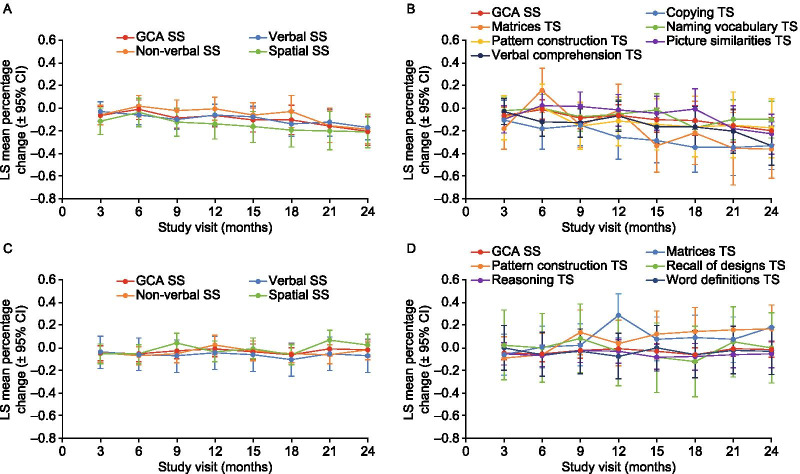
Adjusted LS mean percentage change from baseline in (**A**) DAS-II early years cluster standard scores, (**B**) DAS-II early years subtest scores, (**C**) DAS-II school age cluster standard scores and (**D**) DAS-II school age subtest scores, compared with the DAS-II early years or school age GCA standard score. School age verbal similarities subtest scores (not shown) were highly variable and the model was not able to converge. Individual plots for each subtest score are provided in Additional file 1: Fig. S3. *CI* confidence interval; *DAS-II* Differential Ability Scales, second edition; *GCA* General Conceptual Ability; *LS* least-squares; *SS* standard score; *TS* *T* score

No baseline variables were significantly associated with percentage change from baseline for GCA scores, and therefore none were included in the MMRM model to produce the plots of adjusted least-squares mean percentage change from baseline for comparison of DAS-II subtest and cluster/composite scores. Adjusted least-squares mean percentage changes from baseline in DAS-II early years verbal and spatial cluster and the pattern construction and picture similarities subtest scores followed a similar trend to the early years GCA score, decreasing over the 24 months (Fig. 3A, B, Additional file 1: Fig. S3). Exceptions to this were the early years non-verbal reasoning ability cluster score (which was stable for the first 18 months before decreasing), the early years copying, verbal comprehension and matrices subtest scores (which declined at a greater rate than the early years GCA score) and the naming vocabulary subtest score (which decreased over the first 18 months of observations, but increased at 21 and 24 months).

Adjusted least-squares mean percentage changes from baseline in DAS-II school age cluster scores including verbal, non-verbal reasoning ability and spatial scores followed a similar trend to the school age GCA score, remaining stable over the 24 months (Fig. 3C, D, Additional file 1: Fig. S4). School age subtest scores also followed a similar trend, remaining stable, except for the pattern construction and matrices subtest scores, which improved over 24 months. For the verbal similarities subtest, the scores were highly variable and the model was not able to converge (Additional file 1: Fig. S4F).

Some DAS-II assessments recorded for patients in this study were at the floor (the lowest possible score), but never in all six subtests at the same visit. The most common subtest with floor scores for patients assessed with the early years battery was copying; the most common subtest with floor scores among patients completing the school age battery was verbal similarities.

### VABS-II

**Fig. 4:**
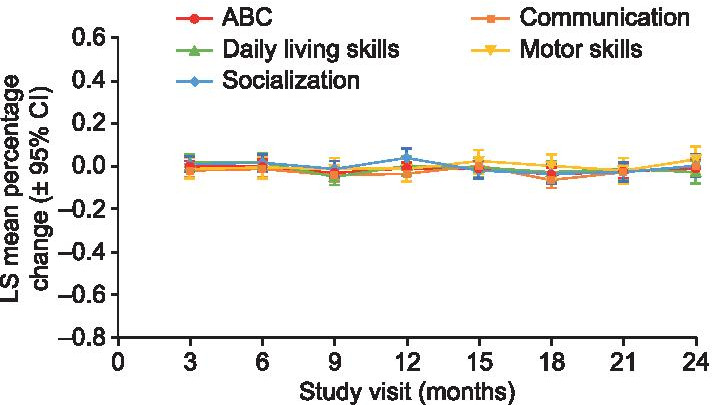
Adjusted LS mean percentage change from baseline in VABS-II ABC and domain scores. *ABC* Adaptive Behavior Composite; *CI* confidence interval; *LS* least-squares; *VABS-II* Vineland Adaptive Behavior Scales, second edition

VABS-II ABC standard scores were stable over the study period, with a mean (SD) score of 81.8 (11.8, n = 36) at baseline and 81.0 (10.2, n = 13) at 24 months. While the mean ABC score was below one SD of the population mean, the ABC scores ranged between 3 SDs below the mean and 1 SD above the mean during the 24-month observation period. Age was the only baseline variable significantly associated with ABC score and, therefore, was included in the MMRM analysis for subdomain and domain scores. Adjusted least-squares mean percentage changes from baseline remained stable over 24 months (domain scores shown in Fig. 4, subdomain scores shown in Additional file 1: Figs S5–S8). An exception was the socialization interpersonal relationships subdomain score, which varied widely over all study visits (Additional file 1: Fig. S8A).

### HS-FOCUS

**Fig. 5:**
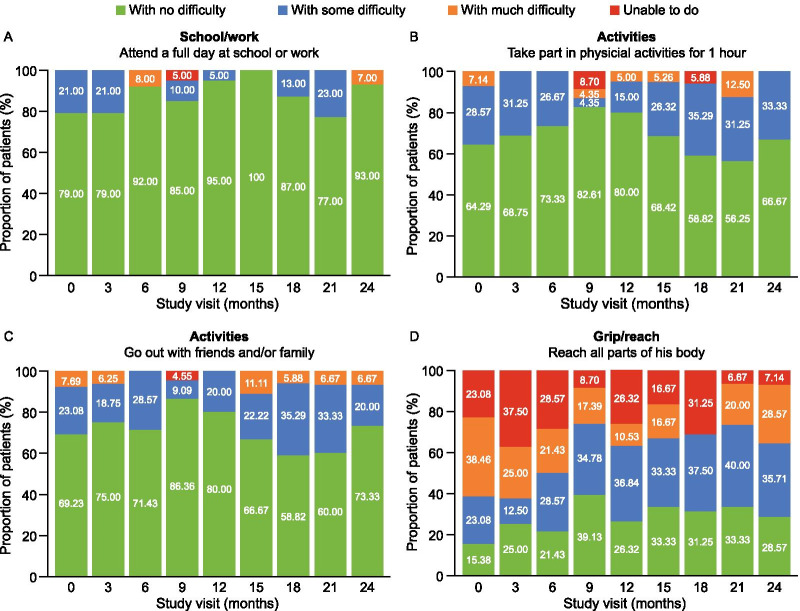
Parent/caregiver responses to HS-FOCUS items assessing ability to (**A**) attend a full day of school or work, (**B**) take part in physical activities for 1 h, (**C**) go out with friends and/or family and (**D**) reach all parts of their body. Data for all HS-FOCUS items are shown in Additional file 1: Figure S9. *HS-FOCUS* Hunter Syndrome-Functional Outcomes for Clinical Understanding Scale

There was no clear pattern of change in HS-FOCUS items over the 24 months of the observational study, with little variability in the responses of parents/caregivers for many of these items. A high functional performance was reported at baseline, with over 50% of caregivers reporting ‘no difficulty’ for 11 of the 21 items. For example, at baseline, 11/14 (79%) of parents/caregivers reported that their children had ‘no difficulty’ attending a full day of school or work, and 3/14 (21%) reported only ‘some difficulty’ (Fig. 5A). Similar high baseline values were reported for other HS-FOCUS items, including the ability to take part in physical activities for 1 h (Fig. 5B), go out with friends and/or family (Fig. 5C), do daily activities without losing breath, talk to someone without losing breath, throw/catch a medium-sized ball, turn pages in a book, walk up a flight of stairs and stand without getting tired for 10 min.

In contrast, items within the grip/reach domain differentiated varying levels of functional ability (Fig. 5D). At baseline, when caregivers were asked whether their children could reach all parts of their body, 2/13 (15.4%), 3/13 (23.1%) and 5/13 (38.5%) reported ‘no difficulty’, ‘some difficulty’ and ‘much difficulty’, respectively, and 3/13 (23.1%) stated that they were unable to reach all parts of their body. Data for all HS-FOCUS items are shown in Additional file 1: Fig S9.

### Associations between DAS-II GCA, VABS-II ABC and HS-FOCUS scores

There was a significant positive association between DAS-II GCA and VABS-II ABC standard scores (β = 0.1104; *p* = 0.0316). For HS-FOCUS, percentage change from baseline could not be evaluated as the outcome variable because values for some items were not available at baseline (e.g. when parents had reported ‘not applicable’ for items such as schoolwork and walking). Therefore, associations between HS-FOCUS and DAS-II GCA and VABS-II ABC scores were investigated using raw scores for each item and domain.

The DAS-II GCA score was significantly (*p* < 0.05) and negatively associated with two of the five domains and five of the 21 items of the HS-FOCUS (Table 3), indicating that increasing cognitive function is associated with better functional abilities. For the VABS-II ABC score, there was a significant (*p* < 0.05) negative association with two domains and three items of the HS-FOCUS, suggesting that increasing adaptive behavior was associated with improved functional performance.

**Table 3:** Associations between DAS-II GCA and VABS-II ABC standard scores and HS-FOCUS items

HS-FOCUS item	DAS-II GCA score coefficient (standard error)	VABS-II ABC score coefficient (standard error)
Activities (domain score)	−0.00714 (0.00364)	−0.01233* (0.00520)
Go out with friends and/or family	−0.00823* (0.00396)	−0.01214* (0.00583)
Take part in physical activities for 1 h	−0.00780 (0.00439)	−0.01337* (0.00614)
Breathing (domain score)	0.00170 (0.00424)	0.00689 (0.00606)
Do daily activities without losing breath	−0.00427 (0.00472)	0.00692 (0.00682)
Sleep without snoring	0.00635 (0.00612)	0.01870* (0.00857)
Talk to someone else without losing breath	0.00171 (0.00421)	−0.00049 (0.00623)
Breathe without making noise at rest	0.00369 (0.00538)	0.00747 (0.00781)
Grip/reach (domain score)	−0.00775** (0.00277)	−0.01074** (0.00411)
Place palm and fingers flat on a table	−0.01190 (0.00611)	−0.00676 (0.00862)
Throw/catch a medium-sized ball	−0.00927* (0.00390)	−0.01107 (0.00557)
Pour a drink into a cup	−0.00688 (0.00559)	−0.01340 (0.00788)
Reach all parts of his body	−0.01549* (0.00702)	−0.00972 (0.00961)
Button a shirt	−0.00832 (0.00672)	−0.01516 (0.00915)
Put on shoes while sitting in a chair	0.00344 (0.00627)	−0.01324 (0.00923)
Touch the top of his head	−0.00838 (0.00530)	−0.00844 (0.00775)
Turn pages in a book	−0.00662* (0.00306)	−0.00616 (0.00443)
School/work (domain score)	−0.01221*** (0.00304)	−0.00853 (0.00452)
Finish all classroom assignments	−0.01551** (0.00495)	−0.00842 (0.00724)
Attend a full day of school or work	−0.00488 (0.00353)	−0.00522 (0.00491)
Walking/standing (domain score)	−0.00163 (0.00230)	−0.00230 (0.00325)
Walk up a flight of stairs	0.00267 (0.00243)	0.00151 (0.00345)
Stand without getting tired for 10 min	0.00055 (0.00337)	0.00067 (0.00489)
Step on a stool	−0.00695 (0.00339)	−0.00116 (0.00493)
Walk without getting tired for 15–20 min	0.00468 (0.00430)	0.00340 (0.00604)
Walk for 20 m (yards) without stopping	0.00531 (0.00507)	−0.00842 (0.00722)

Bold font indicates statistically significant associations and asterisks denote *p* values (*p < 0.05, **p < 0.01, ***p < 0.001). A negative association indicated that increasing cognitive function/adaptive behavior (assessed using DAS-II GCA/VABS-II ABC scores) was associated with improved functional performance (assessed using the HS-FOCUS)

*ABC* Adaptive Behavior Composite; *DAS-II* Differential Ability Scales, second edition; *GCA* General Conceptual Ability; *HS-FOCUS* Hunter Syndrome-Functional Outcomes for Clinical Understanding Scale; *VABS-II* Vineland Adaptive Behavior Scales, second edition

## Discussion

This post hoc analysis examined changes in cognitive function, adaptive behavior and functional outcomes in patients with MPS II and early cognitive impairment over 2 years of follow-up, as assessed using the DAS-II, VABS-II and HS-FOCUS, respectively. The aim of our analysis was to better understand the ability of these instruments to measure changes in patients with neuronopathic MPS II in a natural history setting, and thus inform the analysis and interpretation of data collected using these tools in clinical trials.

The DAS-II has been used to assess cognitive ability in several studies of patients with MPS II and is considered an appropriate tool for measuring the effect of treatment on cognitive outcomes in individuals with the disease in clinical trials [16–18, 25]. The DAS-II GCA score measures cognitive ability relative to a normative sample of same-age, typically developing peers and does not provide information on absolute changes in performance over time [26]. A decrease in an individual’s GCA score may reflect deteriorating cognitive function, stabilization of cognitive development or below-average improvement in cognitive ability [26]. In this analysis, DAS-II early years GCA scores decreased over the 2-year study period, whereas DAS-II school age GCA scores remained stable. These results suggest that the DAS-II was able to measure changes in cognitive function in the younger patients assessed with the early years test battery in this study over 2 years. The different pattern of changes in GCA score over time in the two age groups may have implications for the use of this measure in clinical trial endpoints for investigational treatments. In younger children, stabilization or a smaller decrease in GCA score may be sufficient to demonstrate a treatment effect over at least 2 years, whereas in older children it is possible that gains in GCA score would need to be shown.

To help overcome the challenges associated with interpreting changes in GCA scores and to allow characterization of absolute changes in norm-based ability test scores, the Projected Retained Ability Score (PRAS) was recently developed and retrospectively applied to DAS-II GCA scores in three children with MPS II from this observational study [26]. Among children with relative declines in norm-based GCA scores, the PRAS method was able to distinguish between deteriorating cognitive function, stabilization of cognitive development and below-average improvement in cognitive ability [26]. Mathematical methods have been proposed for evaluating the statistical reliability of differences in GCA/PRAS scores as well as the extent to which they are clinically meaningful [26]. Further research is required to increase our understanding of the validity and clinical applications of PRAS in measuring absolute changes in cognitive function in patients with MPS II.

The content validity of the DAS-II in different populations with MPS II has not yet been fully established and is important if this tool is to be widely used for assessing treatment effects in patients with this condition [27]. It is not yet known whether the DAS-II is equally suitable for younger and older children with greater or lesser degrees of cognitive impairment. Younger children with MPS II may experience an early cognitive decline and then stabilize in terms of developmental gains at a more expected rate as they grow older. It would be valuable to define a more specific age when children with neuronopathic MPS II experience cognitive decline and stabilization, however this was not possible with the data available from this natural history study.

In patients with MPS II and low cognitive function at baseline, the activities of daily living domain from the VABS-II instrument would be expected to decline significantly over time for children older than 6 years of age [8]. However, in this analysis, VABS-II scores remained relatively stable over 2 years. This may be because the VABS-II is not sensitive enough to detect small changes in young children with MPS II and moderately low adaptive behavior at baseline (mean VABS-II ABC standard score of 81.8). Parents/caregivers providing responses for the VABS-II may also be unable to detect small changes after adaptation to their child’s behavior. Changes in adaptive behavior measured by the VABS-II may be more apparent when assessed beyond a 2-year period and could still be a measurement of interest over longer periods of time.

The VABS has been considered an appropriate tool for assessing adaptive behavior in clinical trials evaluating the effect of treatment in children with MPS II [18]. The content validity of the VABS has not been extensively studied in the context of MPS II, however, therefore further research may be needed to confirm whether this is a sufficiently sensitive tool to measure adaptive behavior in these patients [27]. Additional limitations of the VABS include those inherent in an instrument based on parent/caregiver responses, variability of results depending on the administrator, existing parental expectations and other factors that can affect parental observations of the child’s behavior.

There was no clear pattern of change in most HS-FOCUS items over the 2-year study period, and high functional performance was reported for items at baseline. This indicates that the items were not sensitive enough to differentiate between patients with various levels of functional performance, creating a ceiling effect. In contrast, items within the grip/reach domain showed sensitivity in detecting varying levels of functional difficulties in patients with MPS II, possibly owing to the specificity of the questions asked and high number of items included in this domain.

The shortened version of the HS-FOCUS questionnaire used in this study has previously been described as a valid and reliable tool with which to assess the impact of MPS II on physical functioning [23]. However, the activities domain of the HS-FOCUS has a slightly lower test–retest reliability in the shortened version, which contains only two items [23]. Responsiveness to changes in physical functioning and activity limitations was not evaluated during the validation of this tool [23]. In addition, the HS-FOCUS was validated using data from patients with a mean age of 10.8 years [23], whereas most patients in this study were younger than 7 years old at baseline. Some HS-FOCUS items and domains may not be sensitive to early functional decline in very young children with MPS II or may not measure specific disease burden in patients this young. Other limitations of the HS-FOCUS include the lack of objectivity inherent in assessments based on parent/caregiver responses and the fact that caregivers may find it difficult to observe functions for some items, such as snoring while sleeping. In the longer version of the HS-FOCUS, sleep questions were removed [21].

There was a statistically significant association between DAS-II GCA scores and VABS-II ABC standard scores after controlling for the within-patient correlation. DAS-II GCA was significantly negatively associated with seven domains/items of the HS-FOCUS and the VABS II ABC was associated with five domains/items. This indicates that functional performance in patients with MPS II reflects cognitive development and adaptive behavior to some extent. The magnitude of these associations, however, could not be determined from this analysis. Further research into HS-FOCUS items associated with adaptive behavior and cognitive function is required.

Limitations of this analysis included those inherent in post hoc analyses and the small sample size, which was further affected by the loss of patients over time who discontinued involvement in the observational study to enroll in a phase 2/3 study of intrathecally delivered idursulfase. The patient population who remained in the natural history study at 2 years may therefore have had different characteristics from those who originally entered the study. Data were not available for every patient at each visit, which, together with the discontinuation of patients, resulted in missing data values during the study. In addition, patients were followed for 2 years, which may not have been long enough to record changes in all measures. The content validity of the assessment tools used in this analysis for MPS II has also not yet been fully established. Several challenges are associated with assessing cognitive function and adaptive behavior in children with MPS disorders, including difficulties in measuring changes in cognitive function in individuals with neurodegenerative disease, and the interference of sensory, motor and behavioral problems caused by somatic disease manifestations with neurocognitive testing results [28, 29]. Work is ongoing to develop an MPS II-specific neurobehavioral rating scale [30]. An instrument for which measurement is not confounded by inherent physical disabilities in the disease would help clinicians to obtain a more accurate measurement of cognitive abilities in children with MPS II [29].

## Conclusions

Findings from this post hoc analysis provide data to inform the selection of cognitive, behavioral and functional assessment tools for future clinical trials in patients with MPS II. The DAS-II results indicated changes in cognitive ability in younger children assessed with the early years test battery over the 2-year study, whereas cognitive function remained stable in older children assessed with the school age test battery, suggesting that age-specific clinical trial endpoints may be needed. The VABS-II and HS-FOCUS may not be appropriate clinical trial endpoints for measuring behavioral or functional changes over a 2-year period. Further validation studies and novel tools and approaches, such as the PRAS method, may play an important role in improving assessment of cognitive function and behavior in patients with MPS II.

## Supplementary Information

**Additional file 1: Table S1**. Individual patient early years and school age DAS-II GCA scores by study visit; **Fig. S1** Patient study flow; **Fig. S2** Individual early years DAS-II GCA scores for patients with assessments available at baseline and month 24; **Fig. S3** Adjusted LS mean percentage change from baseline in DAS-II early years subtest standard scores compared with the DAS-II early years GCA standard score: (A) matrices, (B) pattern construction, (C) verbal comprehension, (D) copying, (E) naming vocabulary and (F) picture similarities; **Fig. S4** Adjusted LS mean percentage change from baseline in DAS-II school age subtest standard T scores compared with the DAS-II school age GCA standard score: (A) matrices, (B) pattern construction, (C) recall of designs, (D) reasoning, (E) word definitions and (F) verbal similarities; **Fig. S5** Adjusted LS mean percentage change from baseline in VABS-II communication subdomain V-scale scores compared with the VABS-II ABC score: (A) expressive communication, (B) receptive communication and (C) written communication; **Fig. S6** Adjusted LS mean percentage change from baseline in VABS-II daily living skills subdomain V-scale scores compared with the VABS-II ABC score: (A) community daily living skills, (B) domestic daily living skills and (C) personal daily living skills; **Fig. S7** Adjusted LS mean percentage change from baseline in VABS-II motor skills subdomain V-scale scores compared with the VABS-II ABC score: (A) fine motor skills and (B) gross motor skills; **Fig. S8** Adjusted LS mean percentage change from baseline in socialization subdomain V-scale scores compared with the VABS-II ABC score: (A) socialization interpersonal relationships, (B) socialization play and leisure time and (C) socialization coping skills;** Fig. S9** HS-FOCUS domain and item scores: (A) activities, (B) breathing, (C) grip/reach, (D) school/work and (E) walking/standing.

## Data Availability

The datasets, including redacted study protocol, redacted statistical analysis plan, and individual participants' data supporting the results reported in this article, will be made available within 3 months from initial request, to researchers who provide a methodologically sound proposal. The data will be provided after its de-identification in compliance with applicable privacy laws, data protection and requirements for consent and anonymization.

## Abbreviations

ABC: Adaptive Behavior Composite
CI: Confidence interval
DAS-II: Differential Ability Scales, second edition
GCA: General Conceptual Ability
HS-FOCUS: Hunter Syndrome-Functional Outcomes for Clinical Understanding Scale
I2S: Iduronate-2-sulfatase
MMRM: Mixed-effects model for repeated measures
MPS: Mucopolysaccharidosis
PRAS: Projected Retained Ability Score
SD: Standard deviation
VABS-II: Vineland Adaptive Behavior Scale, second edition
