# Supplementary material for: Analysis of cognitive ability and adaptive behavior assessment tools used in an observational study of patients with mucopolysaccharidosis II
Source: Orphanet J Rare Dis. 2021 Dec 4;16:501. doi: 10.1186/s13023-021-02118-3 (PMC8643008; doi:10.1186/s13023-021-02118-3)
Supplement: Supplementary file 1 — Additional file 1: Table S1. Individual patient early years and school age DAS-II GCA scores by study visit; Fig. S1 Patient study flow; Fig. S2 Individual early years DAS-II GCA scores for patients with assessments available at baseline and month 24; Fig. S3 Adjusted LS mean percentage change from baseline in DAS-II early years subtest standard scores compared with the DAS-II early years GCA standard score: (A) matrices, (B) pattern construction, (C) verbal comprehension, (D) copying, (E) naming vocabulary and (F) picture similarities; Fig. S4 Adjusted LS mean percentage change from baseline in DAS-II school age subtest standard T scores compared with the DAS-II school age GCA standard score: (A) matrices, (B) pattern construction, (C) recall of designs, (D) reasoning, (E) word definitions and (F) verbal similarities; Fig. S5 Adjusted LS mean percentage change from baseline in VABS-II communication subdomain V-scale scores compared with the VABS-II ABC score: (A) expressive communication, (B) receptive communication and (C) written communication; Fig. S6 Adjusted LS mean percentage change from baseline in VABS-II daily living skills subdomain V-scale scores compared with the VABS-II ABC score: (A) community daily living skills, (B) domestic daily living skills and (C) personal daily living skills; Fig. S7 Adjusted LS mean percentage change from baseline in VABS-II motor skills subdomain V-scale scores compared with the VABS-II ABC score: (A) fine motor skills and (B) gross motor skills; Fig. S8 Adjusted LS mean percentage change from baseline in socialization subdomain V-scale scores compared with the VABS-II ABC score: (A) socialization interpersonal relationships, (B) socialization play and leisure time and (C) socialization coping skills; Fig. S9 HS-FOCUS domain and item scores: (A) activities, (B) breathing, (C) grip/reach, (D) school/work and (E) walking/standing. [file 13023_2021_2118_MOESM1_ESM.pdf]

Additional file 1

Table S1. Individual patient early years and school age DAS-II GCA scores by study visit

	Study visit (month)								
**Patient**	**0**	**3**	**6**	**9**	**12**	**15**	**18**	**21**	**24**
**#1***	60	64	72	67	-	-	-	-	-
**#2***	87	83	66	75	77	-	-	-	-
**#3***	89	80	63	98	84	80	-	-	-
**#4***	95	87	88	-	74	-	-	-	-
**#5***	83	66	-	-	-	-	-	-	-
**#6**	91	-	77	-	-	-	-	-	-
**#7**	59	-	-	-	-	39	42	32	33
**#8**	70	-	-	-	-	-	-	-	34
**#9***	66	-	-	-	-	-	-	-	-
**#10**	81	82	83	85	89	86	84	83	87
**#11**	62	63	-	73	72	63	68	68	66
**#12**	79	-	-	-	-	-	-	-	-
**#13***	56	46	50	33	45	-	-	-	-
**#14***	90	92	90	84	91	87	71	-	88
**#15***	68	63	65	63	-	-	-	-	-
**#16***	92	87	91	70	77	79	-	-	-
**#17***	-	-	77	64	69	76	56	61	-
**#18**	-	78	86	95	87	87	89	94	92
**#19***	64	63	64	-	-	-	-	-	-
**#20**	55	47	55	51	48	51	47	56	-
**#21**	79	84	84	89	89	82	-	-	-
**#22**	59	42	52	37	-	-	-	-	-
**#23***	65	-	-	-	-	-	-	-	-
**#24***	-	73	-	55	-	-	-	-	-
**#25**	81	80	80	73	77	73	76	76	74
**#26***	-	84	91	86	-	-	-	-	-
**#27***	-	83	-	-	-	-	-	-	-
**#28***	-	-	78	-	-	-	-	-	-
**#29***	63	75	-	-	-	-	-	-	-
**#30***	82	90	90	52	-	-	-	-	-
**#31**	105	76	119	115	113	116	111	115	118
**#32**	55	59	60	71	-	-	-	-	-
**#33**	48	62	69	61	58	44	47	45	37
**#34**	86	84	82	82	78	85	81	81	78
**#35**	75	77	74	81	77	82	77	76	74
**#36**	72	69	74	68	68	73	63	75	71
**#37**	56	51	43	48	51	47	46	49	52
**#38**	65	55	55	55	66	61	65	63	66

Asterisks indicate the 19 patients who discontinued to participate in the phase 2/3 intrathecal idursulfase trial (HGT-HIT-094; ClinicalTrials.gov identifier: NCT02055118). Hyphens denote that data were not available for that visit.

DAS-II, Differential Ability Scales, second edition; GCA, General Conceptual Ability

Fig. S1 Patient study flow

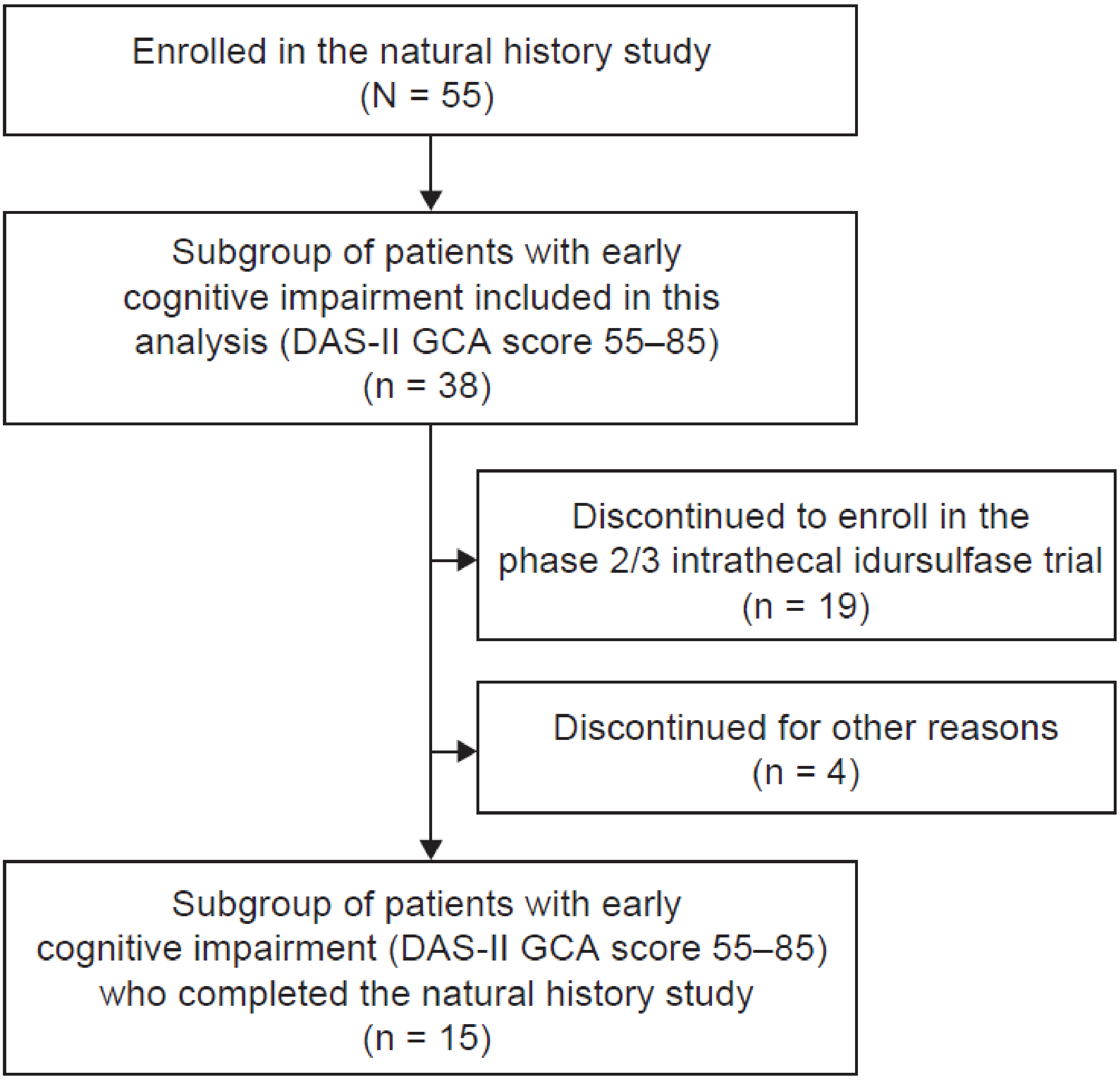

DAS-II, Differential Ability Scales, second edition; GCA, General Conceptual Ability

Fig. S2 Individual early years DAS-II GCA scores for patients with assessments available at baseline and month 24

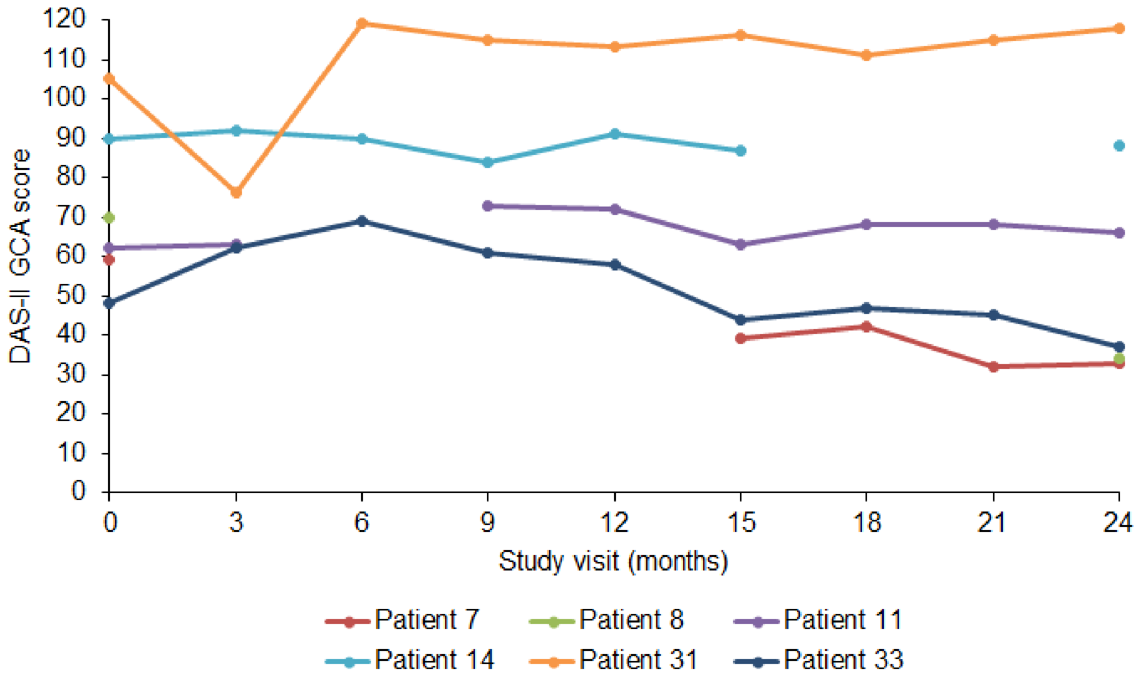

DAS-II, Differential Ability Scales, second edition; GCA, General Conceptual Ability

Fig. S3 Adjusted LS mean percentage change from baseline in DAS-II early years subtest standard scores compared with the DAS-II early years GCA standard score: (A) matrices, (B) pattern construction, (C) verbal comprehension, (D) copying, (E) naming vocabulary and (F) picture similarities

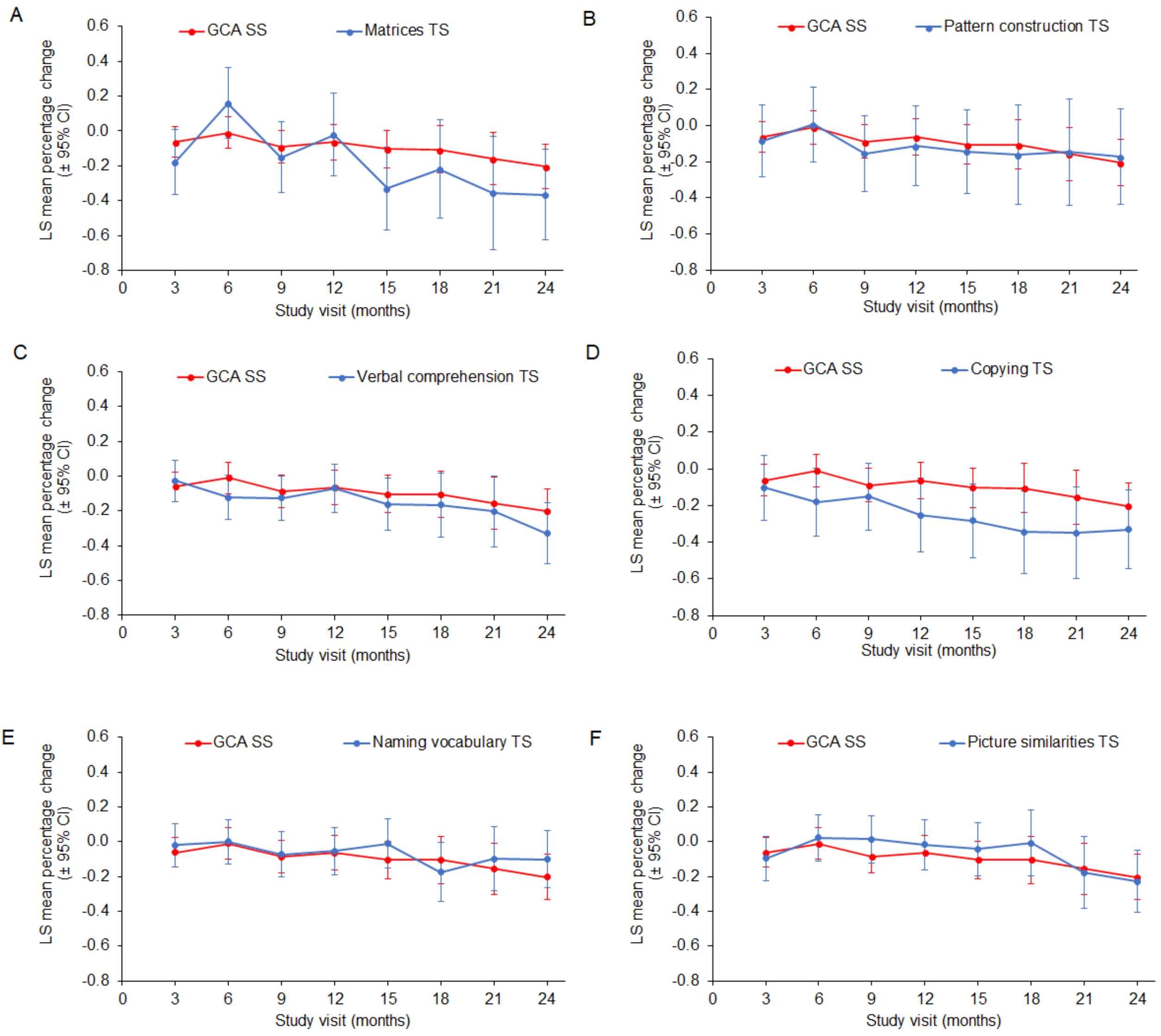

CI, confidence interval; DAS-II, Differential Ability Scales, second edition; GCA, General Conceptual Ability; LS, least-squares; SS, standard score; TS, *T* score

Fig. S4 Adjusted LS mean percentage change from baseline in DAS-II school age subtest standard *T* scores compared with the DAS-II school age GCA standard score: (A) matrices, (B) pattern construction, (C) recall of designs, (D) reasoning, (E) word definitions and (F) verbal similarities

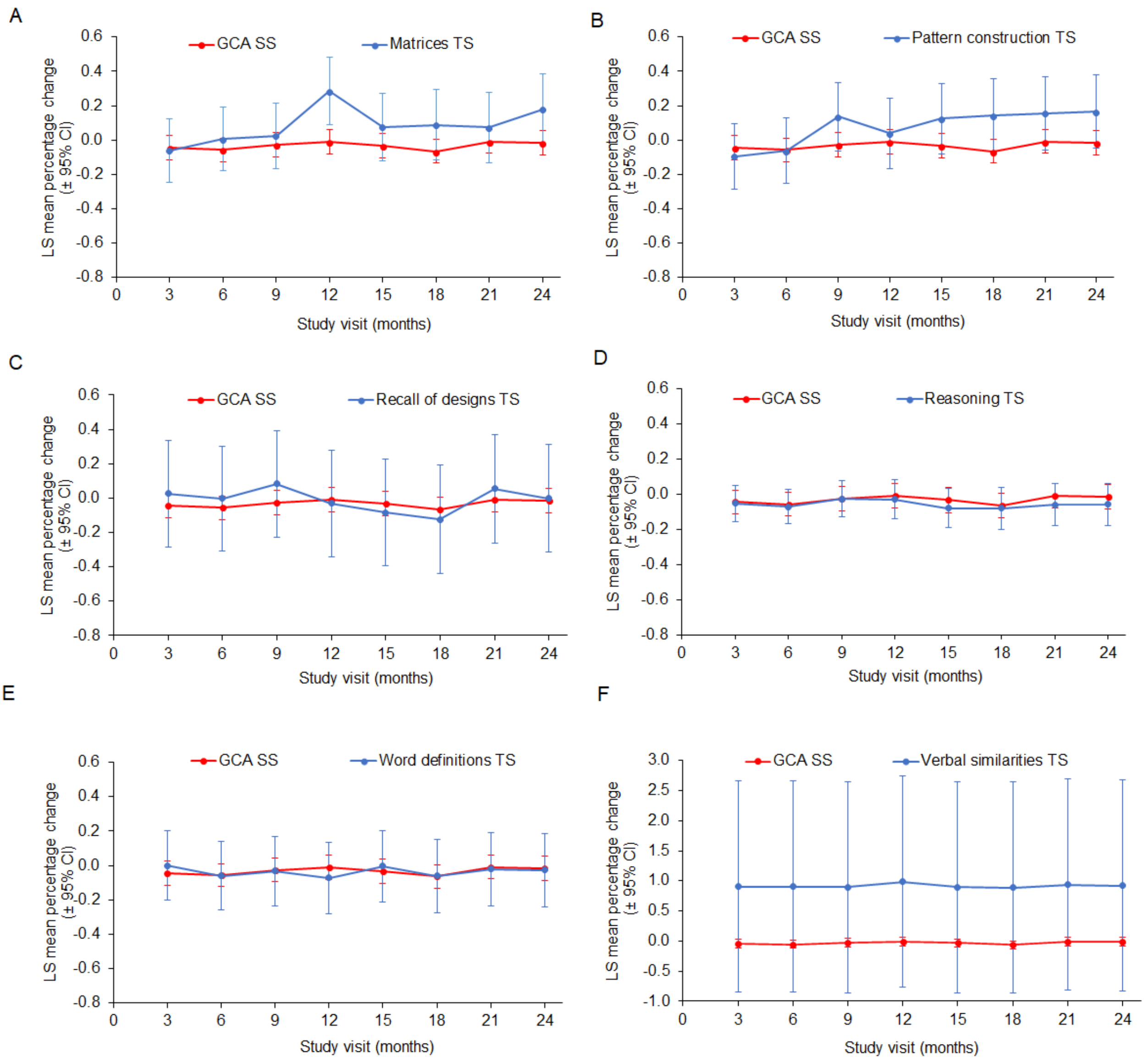

CI, confidence interval; DAS-II, Differential Ability Scales, second edition; GCA, General Conceptual Ability; LS, least-squares; SS, standard score; TS, *T* score

Fig. S5 Adjusted LS mean percentage change from baseline in VABS-II communication subdomain V-scale scores compared with the VABS-II ABC score: (A) expressive communication, (B) receptive communication and (C) written communication

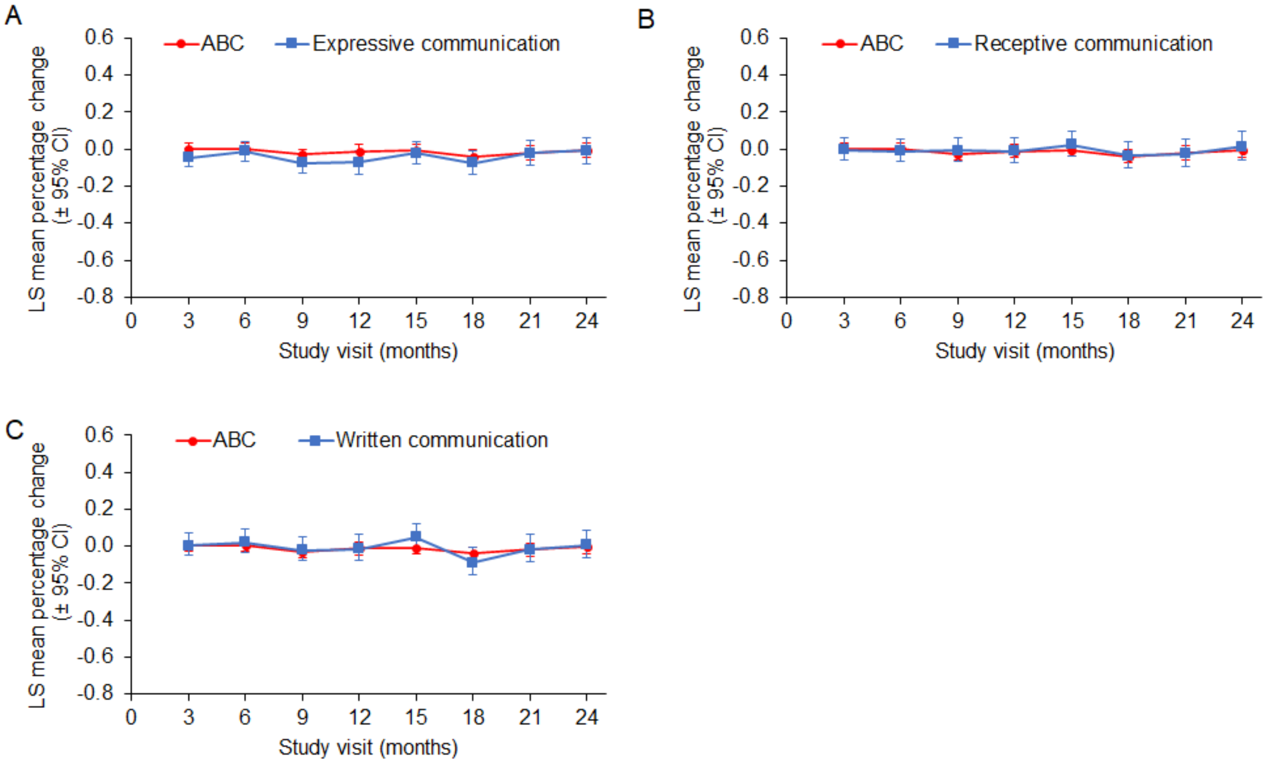

ABC, Adaptive Behavior Composite; CI, confidence interval; LS, least-squares; VABS-II, Vineland Adaptive Behavior Scales, second edition

Fig. S6 Adjusted LS mean percentage change from baseline in VABS-II daily living skills subdomain V-scale scores compared with the VABS-II ABC score: (A) community daily living skills, (B) domestic daily living skills and (C) personal daily living skills

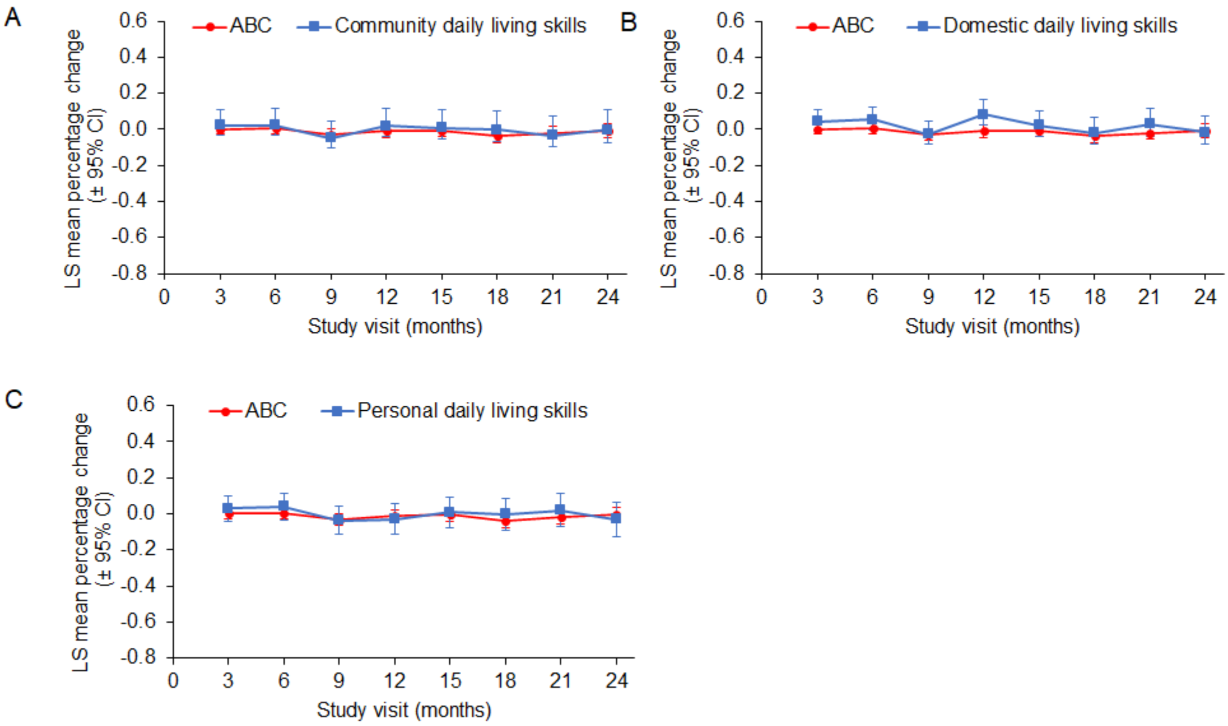

ABC, Adaptive Behavior Composite; CI, confidence interval; LS, least-squares; VABS-II, Vineland Adaptive Behavior Scales, second edition

Fig. S7 Adjusted LS mean percentage change from baseline in VABS-II motor skills subdomain V-scale scores compared with the VABS-II ABC score: (A) fine motor skills and (B) gross motor skills

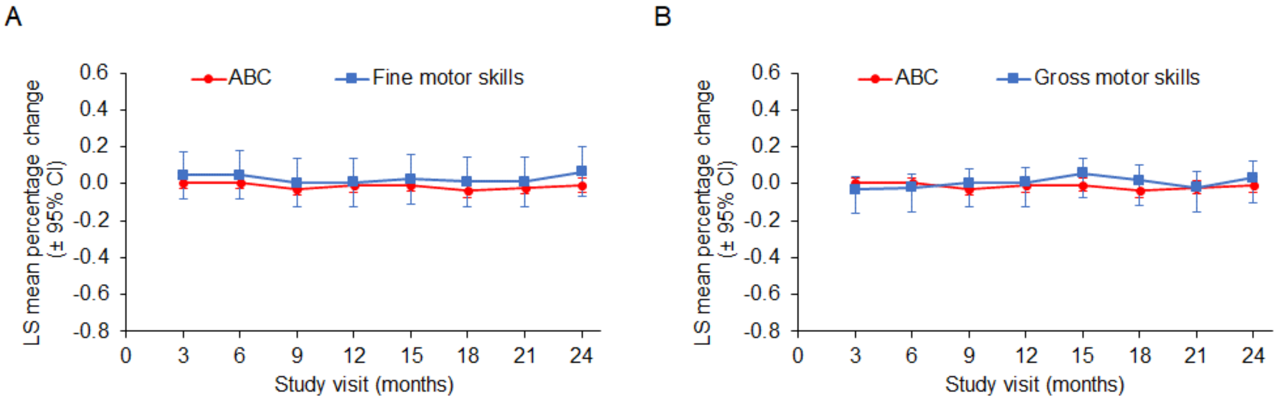

ABC, Adaptive Behavior Composite; CI, confidence interval; LS, least-squares; VABS-II, Vineland Adaptive Behavior Scales, second edition

Fig. S8 Adjusted LS mean percentage change from baseline in socialization subdomain V-scale scores compared with the VABS-II ABC score: (A) socialization interpersonal relationships, (B) socialization play and leisure time and (C) socialization coping skills

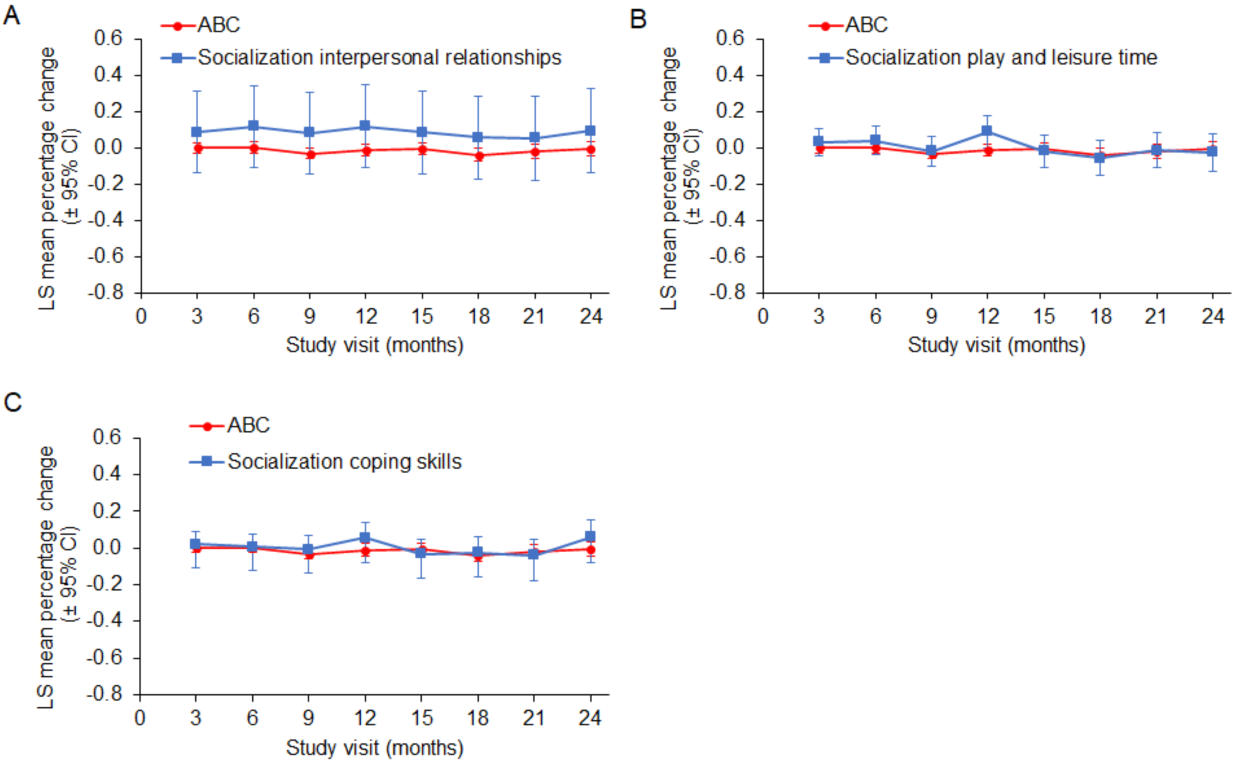

ABC, Adaptive Behavior Composite; CI, confidence interval; LS, least-squares; VABS-II, Vineland Adaptive Behavior Scales, second edition

Fig. S9 HS-FOCUS domain and item scores: (A) activities, (B) breathing, (C) grip/reach, (D) school/work and (E) walking/standing

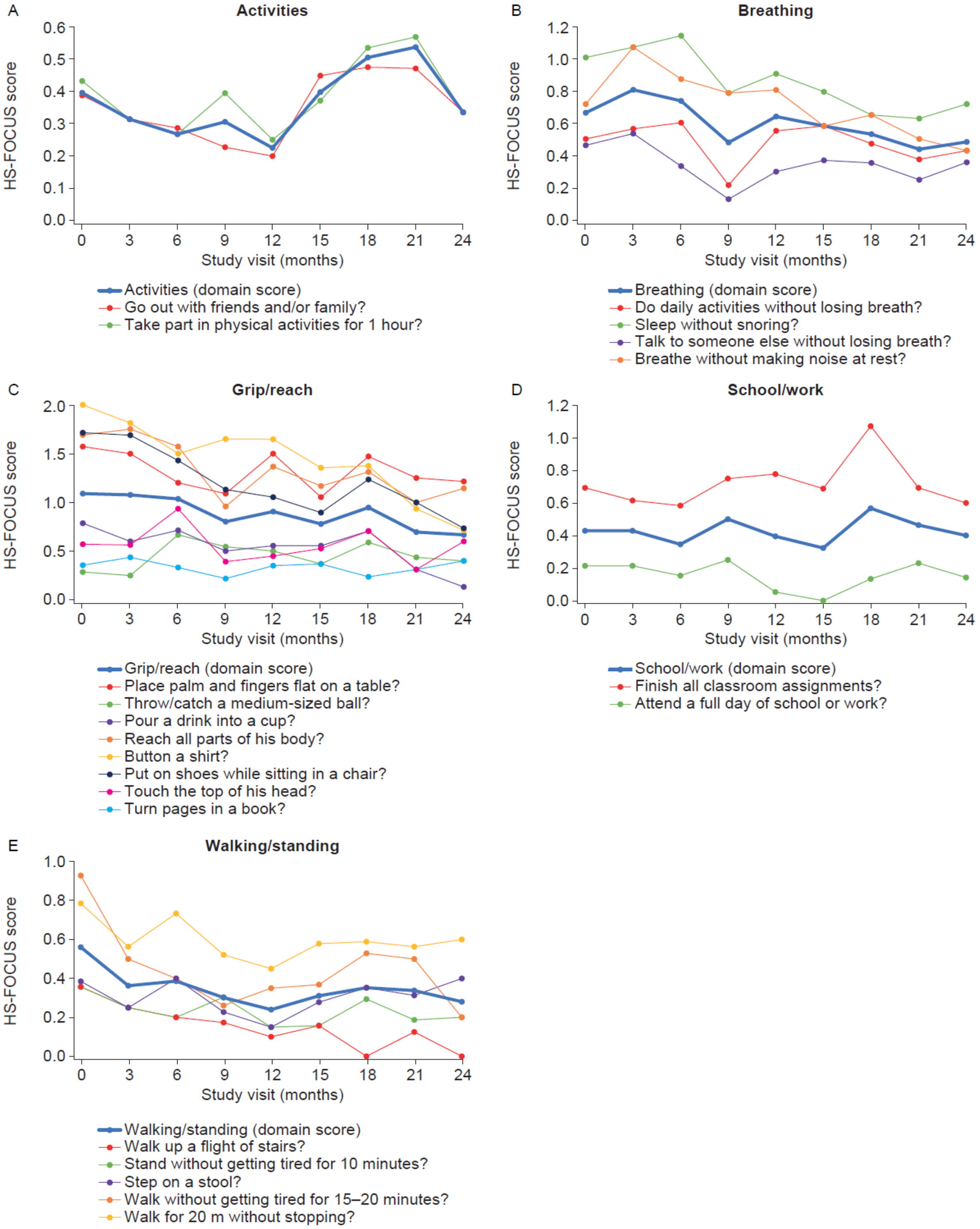

HS-FOCUS, Hunter Syndrome-Functional Outcomes for Clinical Understanding Scale
